# Capacity and consent to treatment – how well did we do?

**DOI:** 10.1192/bjo.2021.829

**Published:** 2021-06-18

**Authors:** Khui Chiang Wee, Nithya Anandan, Nguemo Angahar, Abhilash Mannam

**Affiliations:** Northwest Boroughs HealthCare NHSFT

## Abstract

**Aims:**

An audit on capacity assessment and consent to treatment on inpatient visits to Atherleigh Park Hospital was performed using the Mental Health Act Code of Practice as a framework. Six standards were evaluated:

1) documentation of capacity assessment in patient care records

2) documentation of patients who display a lack of capacity

3) completion of a Section 58 and/or 62 for detained patients

4) documentation of medicines on T2/T3 form and if they match with the patient's prescription chart

5) evidence of medication concordance and monitoring of adverse side effects

6) patient education on medicines prescribed for them

**Method:**

Inclusion criteria included patients who were detained under Sections 2, 3 and informal admissions, who were admitted for 72 hours or more, between October and December 2019. This gave a total sample size of 75. Data were collected by looking at patients’ care records and if applicable, their Section paperwork to identify any documentations related to the standards evaluated as above. Data collected were transcribed to a web link, downloaded and analysed.

**Result:**

In standard 1), it was found that 77% of the capacity assessment and consent to treatment forms were recorded in patient care records. Of these, 100% of were completed by a medic and 99% of all sections in the form were completed. However, only 57% of patients were re-assessed when their capacity and consent changed during admission. In standards 2), 3) and 4), documentation of patients who lacked capacity, completion of a Section 58 or 62 form and charting of medications on the T2/T3 forms were fully compliant. In standards 5) and 6), 76% of medication concordance were documented in patients’ records. Only 39% of adverse effects from medications were documented but monitoring compliance was 100%. Medication counselling was done infrequently, with 47% of patients given a leaflet and 28% educated on their side effects.

**Conclusion:**

Action plans were identified. Firstly, to link the capacity assessment form with patient electronic ward round notes to ensure clinicians complete it at the end of a review. In order to monitor adverse effects from medications, physical examination, blood tests and ECG are to be done following a new prescription, and to be repeated if indicated. Information leaflets on common psychiatric medications are to be made readily available for patients. The findings from this service evaluation and the actions plans were shared with doctors. A re-audit is vital to re-evaluate the hospital's compliance.

